# Mammographic microcalcifications and breast cancer tumorigenesis: a radiologic-pathologic analysis

**DOI:** 10.1186/s12885-015-1312-z

**Published:** 2015-04-22

**Authors:** Madiha Naseem, Joshua Murray, John F Hilton, Jason Karamchandani, Derek Muradali, Hala Faragalla, Chanele Polenz, Dolly Han, David C Bell, Christine Brezden-Masley

**Affiliations:** 1 Department of Hematology/Oncology, St. Michael’s Hospital, 30 Bond Street, Toronto, Ontario M5B 1W8 Canada; 2 Faculty of Medicine, University of Toronto, 1 Kings College Circle, Toronto, ON M5S 1A8 Canada; 3 Horizon Health Network, The Moncton Hospital, 135 MacBeath Avenue, Moncton, New Brunswick E1C 6Z8 Canada; 4 Dana-Farber Cancer Institute, Brigham and Women’s Hospital, and Harvard Medical School, 450 Brookline Avenue, Boston, MA 02215 USA; 5 Department of Laboratory Medicine and Pathology, St. Michael’s Hospital, 30 Bond Street, Toronto, ON M5B 1W8 Canada; 6 Department of Medical Imaging, St. Michael’s Hospital, 30 Bond Street, Toronto, ON M5B 1W8 Canada

**Keywords:** Microcalcifications, Breast imaging, Mammography, Tumorigenesis, Breast pathology, HER-2

## Abstract

**Background:**

Microcalcifications (MCs) are tiny deposits of calcium in breast soft tissue. Approximately 30% of early invasive breast cancers have fine, granular MCs detectable on mammography; however, their significance in breast tumorigenesis is controversial. This study had two objectives: (1) to find associations between mammographic MCs and tumor pathology, and (2) to compare the diagnostic value of mammograms and breast biopsies in identifying malignant MCs.

**Methods:**

A retrospective chart review was performed for 937 women treated for breast cancer during 2000–2012 at St. Michael’s Hospital. Demographic information (age and menopausal status), tumor pathology (size, histology, grade, nodal status and lymphovascular invasion), hormonal status (ER and PR), HER-2 over-expression and presence of MCs were collected. Chi-square tests were performed for categorical variables and t-tests were performed for continuous variables. All p-values less than 0.05 were considered statistically significant.

**Results:**

A total of 937 patient charts were included. About 38.3% of the patients presented with mammographic MCs on routine mammographic screening. Patients were more likely to have MCs if they were HER-2 positive (52.9%; p < 0.001). There was a significant association between MCs and peri-menopausal status with a mean age of 50 (64%; p = 0.012). Patients with invasive ductal carcinomas (40.9%; p = 0.001) were more likely to present with MCs than were patients with other tumor histologies. Patients with a heterogeneous breast density (p = 0.031) and multifocal breast disease (p = 0.044) were more likely to have MCs on mammograms. There was a positive correlation between MCs and tumor grade (p = 0.057), with grade III tumors presenting with the most MCs (41.3%). A total of 52.2% of MCs were missed on mammograms which were visible on pathology (p < 0.001).

**Conclusion:**

This is the largest study suggesting the appearance of MCs on mammograms is strongly associated with HER-2 over-expression, invasive ductal carcinomas, peri-menopausal status, heterogeneous breast density and multifocal disease.

## Background

Breast cancer is the most common cancer in females over the age of 20. Breast cancer represents 26% of all newly diagnosed cancer cases in women and 14% of women are is expected to die from it [1]. The incidence rates of breast cancer have risen from 1982 through the early 1990s, in part due to increased mammography screening.

**Figure 1:**
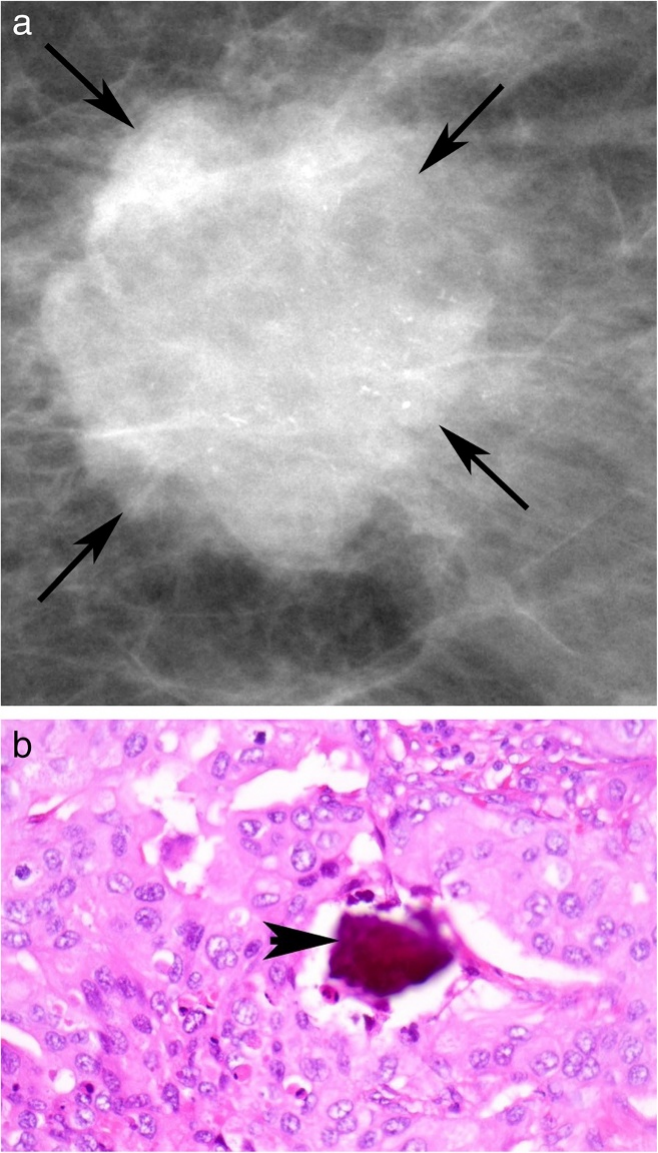
Mammogram and pathology report for HER-2 positive patient. **a**: Digital Mammogram *(Mammomat Novation, Siemens Healthcare, Erlangen, Germany)* of the left breast from a 40 year old woman with a HER-2 positive invasive ductal carcinoma, shows a malignant appearing mass (arrows) with numerous pleomorphic calcifications confined to the mass, BI-RADS 5. **b**: Hematoxyln and eosin stained section (400×) of poorly differentiated invasive ductal carcinoma with microcalcification (arrowhead).

The advent of mammographic screening has not only provided us with the ability to detect potentially fatal tumors at a non-palpable stage, but it has also created the platform to study the natural history of breast cancer in its early stages of development [2]. One of the easily detectable mammographic anomalies, and often the earliest signs of a malignant breast disease, are tiny deposits of calcium in the breast soft tissue, called microcalcifications (MCs) [3]. The presence of MCs was first reported in 1913 by a German surgeon, Solomon, who conducted a radiographic examination of a mastectomy specimen. In 1951, a radiologist named Leborgne proposed that MCs could be the only mammographic manifestation of breast carcinoma [4]. Since then, active efforts have been made by radiologists to identify MCs in mammograms (Figure 1b), making them one of the most important diagnostic markers of breast lesions [5].

Although MCs are also associated with benign conditions such as secretory diseases and fat necrosis, around 40% of breast cancers present with MCs and frequently, serve as the only mammographic features indicating the presence of a tumor [6]. X-ray diffraction and electron microscopic analysis have revealed two distinct forms of MCs based on their appearance and chemical composition [7]. Type I MCs are calcium oxalate crystals, while Type II MCs are composed of another bone specific mineral called hydroxyapatite [3]. Among the two, Type II MCs are exclusively found in malignant breast disease, and these crystals are known to accelerate the pathological process involved in breast cancer.

Malignant MCs have one of three appearances: crushed stone (pleomorphic), powdery, or casting-type [8]. Previous studies have shown that patients presenting with casting-type MCs have aggressive tumor pathology, with a death rate five times that of patients who do not present with such MCs [9].

Considerable progress has been made in understanding the molecular foundations of breast carcinogenesis. However, the biogenesis of MCs and their role in breast cancer is still understudied. MCs are shown to be associated with the overexpression of Human Epidermal Growth Factor Receptor Type 2, HER-2, a transmembrane protein receptor which serves as an independent poor prognostic factor in premalignant breast lesions. Previous studies have also examined the associations between MCs and other prognostic factors of breast cancer, such as Estrogen (ER) and Progesterone receptor (PR) positivity. However, there is currently no consensus on the prognostic significance of MCs in early breast cancer.

This paper presents associations between benign and malignant mammographic MCs, and breast biomarkers, patient demographics, and breast radiological features. It also evaluates the utility of mammograms in identifying MCs by comparing breast biopsy and mammogram reports.

## Methods

### Ethics

Institutional research ethics board approval from St. Michael’s Hospital was obtained for this research study.

### Inclusion/exclusion criteria

All patients seen by medical oncologists at St. Michael’s Hospital Medical Day Care Unit, diagnosed only with invasive breast cancer were included in the study. Benign and malignant microcalcifications on mammography and pathology for patients with invasive breast cancer were included. Patients with non-invasive diseases were not included, as this study’s focus is on investigating the role of MCs in invasive disease. Patients were selected based on availability of electronic health records, dating back to 2000.

### Data acquisition

A retrospective chart review was performed for 937 women treated for breast cancer during 2000–2012 at St. Michael’s Hospital, Toronto, Canada. Demographic information (age and menopausal status), tumor pathology (size, histology, grade, nodal status and lymphovascular invasion), hormonal status (ER and PR), HER-2 overexpression and presence of both benign and malignant MCs on mammograms and pathology reports were collected for breast cancer patients. Mammograms were obtained from the Department of Medical Imaging at St. Michael’s Hospital, using the Digital Mammogram using technology from Siemens Mammomat Novation DR (2004).

### Immunohistochemistry

Hormone receptor status that was collected from the pathology reports, was determined using immunohistochemistry (IHC). ER and PR were detected with the Ventana 6 F11 and Ventana 16 clones, respectively, with heat retrieval pretreatment and no dilution. HMK was detected by using the Dako 34BetaE12 (reacts with cytokeratins 1,5,10,14) with heat retrieval pretreatment and a 1:0 dilution. As per the 2010 College of American Pathologists guidelines, ≥ 1% of tumor cell nuclei must be immunoreactive to be considered ER/PR positive. The same criteria has been used in previous studies. HER-2 was detected using the Novocastra CB11 with a 1:40 dilution. For each antibody used, appropriate second antibodies were complexed to streptavidin and chromagen. IHC is used first for overexpression of HER-2 in genecopy ratio. As per College of American Pathologists 2013 guidelines, any case with a 2+ score on IHC is sent for in situ hybridization, whether fluorescent in situ hybridization (FISH) or bright field dual in situ hybridization (DISH). IHC scores (0) and (1+) are considered negative and nothing else needs to be done. IHC score (2+) is equivocal and needs in situ hybridization. IHC score (3+) is considered positive and nothing else needs to be done. These guidelines were applied to obtain study samples.

### Microcalcifications on pathology

At St. Michael’s Hospital, Stereotactic core biopsy for microcalcifications was obtained, and initially cut into 10 levels from each core and every other level was stained. If MCs were found on the initial levels, nothing more was done. If MCs were not found, the slides were polarized to find polarizable calcium crystals. If MCs were not found, the blocks were further x-rayed and then cut on deeper levels with MCs until they were discovered. The radiology report was also checked as x-ray specimen indicate if there are MCs within the cores submitted. If MCs were found in the specimen radiograph and not in the blocks, examination of deeper levels was conducted to assess as much tissue as possible.

For this study, pathological reports were prepared by staff pathologists, and included if available on patient’s electronic health record.

### Statistical analysis

Descriptive statistics were calculated for each variable of interest. Proportions and frequencies were calculated for categorical variables while means and standard deviations were calculated for continuous variables.

The distribution of the presence of MCs on mammography was examined. Chi square tests were performed to test for associations between the presence of MCs on mammography and categorical variables, while t-tests were performed to test for associations for continuous variables.

The distribution of the presence of both benign and malignant MCs on pathology was examined. The presence of MCs on mammograms was tested for association with the over-expression of HER-2, and hormonal status of ER and PR. All tests were two-sided and p-values less than 0.05 were considered statistically significant. No corrections for multiple testing were done for this exploratory analysis.

## Results

A total of 937 charts were reviewed for patients with stages I-III breast cancer during 2000–2012 at St. Michael’s Hospital, Toronto. Table 1 presents patient characteristics for the twelve variables of interest. About 38.3% of the patients had MCs present of any type, either benign or malignant. The mean age was 58.1 years (age range 25–98 years) with most patients having either ductal (81.3%) or lobular (9.5%) lesions; Of these, only 21.4% of patients had evidence of lymphovascular invasion. In total, 78.2% were ER positive while 64.9% were PR positive. Only 16.3% of the patients were HER-2 positive.

**Table 1:** **List of patient characteristics**

Patient characteristics	n (%)	Mean (±SD)
Age		58.1 (13.3)
Tumor Size		2.5 (1.9)
Mammography Calcifications		
Yes	*287 (38.3)*	
No	*462 (61.7)*	
Recurrence		
Yes	*40 (7.9)*	
No	*466 (92.1)*	
Histology		
Ductal	*738 (81.3)*	
Lobular	*86 (9.5)*	
Other	*84 (9.3)*	
Lymphovascular Invasion		
Yes	*156 (21.4)*	
No	*574 (78.6)*	
Node		
Positive	*274 (34.3)*	
Negative	*526 (65.8)*	
Tumor Grade		
1	*221 (25.3)*	
2	*375 (43.0)*	
3	*277 (31.7)*	
Density		
Almost entirely fatty	*252 (56.0)*	
Scattered	*72 (16.0)*	
Very dense	*17 (3.8)*	
Extremely dense	*24 (5.3)*	
Heterogeneously dense	*57 (12.3)*	
Other	*28 (6.2)*	
Bilaterality		
Yes	*18 (2.9)*	
No	*599 (97.1)*	
Architectural Distortion		
Yes	*90 (17.0)*	
No	*438 (83.0)*	
Focality		
Unifocal	*464 (81.7)*	
Multifocal/Multicentric	*104 (18.3)*	
Menopausal Status		
Pre	*229 (26.1)*	
Peri	*29 (3.3)*	
Post	*620 (70.6)*	
Diabetes		
Yes	*51 (8.5)*	
No	*548 (91.5)*	
Family History of Breast Cancer		
Yes	*158 (35.1)*	
No	*282 (64.1)*	
Children		
Yes	*277 (59.0)*	
Yes-1st pregnancy ≥ 30 years	*53 (11.7)*	
Nulliparous	*124 (27.3)*	
HER-2		
Positive	*139 (16.3)*	
Negative	*713 (83.4)*	
ER		
Positive	*712 (78.2)*	
Negative	*199 (21.8)*	
PR		
Positive	*591 (64.9)*	
Negative	*319 (35.1)*	

This table outlines the proportion (n) of study patients with certain demographic, tumor pathologic, and mammographic characteristics. N = Number of patients in the sample, SD = Standard Deviation.

Table 2 presents the results of the tests of association between the presence of MCs and the other variables of interest. Variable names appearing in bold had a significant association with the presence of MCs.

**Table 2:** **Statistical associations for the presence of MCs on mammography**

(a) Categorical variables	Microcalcifications				Test statistic	
	No		Yes			
	n	(%)	n	(%)	χ^2^	p-value
Recurrence					1.28	*0.258*
Yes	*21*	*(52.5)*	*19*	*(47.5)*		
No	*287*	*(61.6)*	*179*	*(38.4)*		
**Histology**					**15.1**	***0.001*** *
Ductal	*357*	*(59.1)*	*247*	*(40.9)*		
Lobular	*58*	*(82.9)*	*12*	*(17.1)*		
Other	*44*	*(63.8)*	*25*	*(36.2)*		
Lymphovascular Invasion					1.98	*0.159*
Yes	*72*	*(57.6)*	*53*	*(42.4)*		
No	*334*	*(64.9)*	*181*	*(35.1)*		
Node Status					0.08	*0.782*
Positive	*134*	*(62.3)*	*81*	*(37.7)*		
Negative	*287*	*(63.8)*	*163*	*(36.2)*		
Tumor Grade					5.74	*0.057*
1	*133*	*(69.3)*	*59*	*(30.7)*		
2	*192*	*(60.2)*	*127*	*(39.8)*		
3	*122*	*(58.7)*	*86*	*(41.3)*		
**Density**					**12.32**	***0.031***
Almost entirely fatty	*164*	*(65.1)*	*88*	*(34.9)*		
Scattered	*44*	*(61.1)*	*28*	*(38.9)*		
Very dense	*10*	*(58.8)*	*7*	*(41.2)*		
Extremely dense	*16*	*(66.7)*	*8*	*(33.3)*		
Heterogeneously dense	*23*	*(40.4)*	*34*	*(59.7)*		
Other	*17*	*(60.7)*	*11*	*(39.3)*		
Bilaterality					0.18	*0.669*
Yes	*6*	*(50.0)*	*6*	*(50.0)*		
No	*320*	*(60.4)*	*210*	*(39.6)*		
Architectural Distortion					0.81	*0.369*
Yes	*47*	*(56.0)*	*37*	*(48.1)*		
No	*268*	*(61.9)*	*165*	*(38.1)*		
**Focality**					**4.04**	***0.044***
Multifocal/Multicentric	*49*	*(52.1)*	*45*	*(47.9)*		
Unifocal	*271*	*(63.9)*	*153*	*(36.1)*		
**Menopausal Status**					**8.86**	***0.012***
Pre	*111*	*(59.0)*	*77*	*(41.0)*		
Peri	*9*	*(36.0)*	*16*	*(64.0)*		
Post	*331*	*(64.1)*	*185*	*(35.9)*		
Diabetes					0.00	*0.999*
Yes	*26*	*(60.5)*	*17*	*(39.5)*		
No	*294*	*(60.1)*	*195*	*(39.9)*		
Family History					1.66	*0.198*
Yes	*91*	*(56.5)*	*52*	*(43.5)*		
No	*144*	*(63.6)*	*111*	*(36.4)*		
Children					4.33	*0.115*
Yes	*156*	*(62.7)*	*93*	*(37.3)*		
Yes-after age 30	*25*	*(50.0)*	*25*	*(50.0)*		
No	*59*	*(53.6)*	*51*	*(46.4)*		
**HER-2**					**12.9**	***<0.001***
Positive	*48*	*(47.1)*	*54*	*(52.9)*		
Negative	*399*	*(66.2)*	*204*	*(33.8)*		
ER					0.36	*0.549*
Positive	*89*	*(59.7)*	*60*	*(40.3)*		
Negative	*375*	*(62.8)*	*222*	*(37.2)*		
PR					1.42	*0.233*
Positive	*150*	*(59.1)*	*104*	*(40.9)*		
Negative	*314*	*(63.8)*	*178*	*(34.9)*		
(b) Continuous Variables	Microcalcification				Test Statistic	
	No		Yes			
	Mean	SD	Mean	SD	t	p-value
Age	*59.0*	*(13.2)*	*57.4*	*(12.3)*	1.67	*0.100*
Tumor Size	*2.2*	*(1.8)*	*2.5*	*(2.1)*	−1.58	*0.115*

This table outlines statistical associations between the presence of MCs and other variables of interest. Chi square values and p-values are outlines. P-values < 0.05 is considered statistically significant and highlighted in bold.

### Tumor pathology

The relationship between the presence of MCs and histology was significant, (p =0.001). Patients with ductal carcinoma were more likely to have MCs than were patients with other tumor classifications (mammary, lobular, mixed). There was no significant relationship between MCs and lymphovascular invasion or nodal status. Among patients with a grade III tumor, 41.3% had MCs, as opposed to 39.8% with a grade II tumor and 30.7% with a grade I tumor. There was a positive correlation between the presence of MCs and an increase in tumor grade, however, this relationship was not statistically significant (p = 0.057). There was no significant association between the presence of MCs and mean tumor size or the rate of tumor recurrence. Recurrence was measured using a 5 year recurrence end-point, and there was no statistical association between having MCs on mammography and recurrence pattern (p = 0.258).

### Breast biomarkers

**Figure 2:**
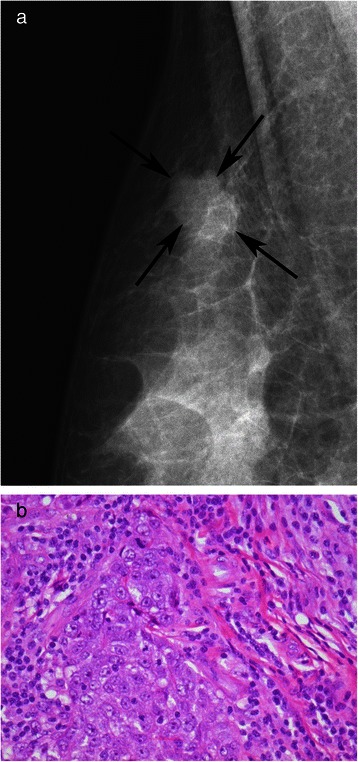
Mammogram and pathology report for HER-2 negative patient. **a**: Digital Mammogram *(Mammomat Novation, Siemens Healthcare, Erlangen, Germany)* of the right breast from a 53 year old woman with HER-2 negative invasive ductal carcinoma, shows a lobulated mass (arrows) in the upper right breast, with no evidence of calcifications, BI-RADS 4. **b**: 400× H&E stained section of invasive ductal carcinoma of no special type composed of pleomorphic, mitotically active ductal epithelial cells with sheet-like growth.

Patients were more likely to have MCs if they had an over-expression of HER-2 (52.9%; p = 0.001). Images from a patient with overexpression of HER-2 showed the presence of MCs in both mammographic images (Figure 1a) and the corresponding pathology sample (Figure 1b). Conversely, neither the mammogram (Figure 2a) nor the pathology sample (Figure 2b) displayed evidence of MCs for patient with HER-2 negative disease. There was no significant association between the presence of MCs with ER (p = 0.549) and/or PR status (p = 0.233).

### Patient demographics

The mean age of pre-menopausal patients was 43.0 while the mean age of peri-menopausal and post-menopausal patients was 50.0 and 64.2 respectively. Menopausal status was recorded on the patient charts, and the age range of all patients was 25–98 years. The relationship between menopause and the presence of MCs was statistically significant (p = 0.012). Among the peri-menopausal patients, 64% had MCs present as opposed to the 41% of pre-menopausal and 35.9% of post-menopausal patients who had MCs. There was a higher likelihood of women to have MCs if they either had no children (43.5%) or had children after age 30 (50%). However, this relationship was not statistically significant (p = 0.115). There was no significant association between MCs and mean age, family history of breast cancer, or diabetes.

### Mammographic characteristics

Patients with heterogeneously dense breasts, as reported on mammograms were more likely to have MCs than all other breast densities (almost entirely fatty, scattered, very dense, extremely dense, other) (p =0.031). Patients with multifocal or multicentric breast cancers were more likely to present with MCs (p = 0.044). There was no significant association between the presence of MCs with architectural distortion within the breast tissue (p = 0.369), or having bilateral breast cancer (p = 0.669).

### MCs on pathology

From the total cohort of 937 patients, there were only 472 patients with MCs noted on pathology. Of these, 52.2% of patients who did not have MCs appearing on mammography had detectable MCs on pathology samples, which were statistically significant (Table 3) (p < 0.001).

**Table 3:** **Sensitivity of mammograms in detecting MCs in comparison to MCs identified in biopsy specimens**

Categorical variables	Microcalcifications (Pathology)				Test statistic	
	No		Yes			
	n	(%)	n	(%)	χ^2^	p-value
Microcalcifications (Mammography)					**32.4**	***<0.001***
No	*134*	*(47.8)*	*147*	***(52.2)***		
Yes	*41*	*(21.4)*	*150*	*(78.5)*		

Bolded numbers indicate statistical significance. Of the 472 patients who had pathology samples available, 147 (31%) had a false negative result, where MCs were detected in pathology samples but not in mammography.

## Discussion

Mammographic MCs serve as important diagnostic markers of benign and malignant breast lesions. However, there is still a lack of consensus regarding the genesis of MCs and their relationship with breast cancer pathology. Our study indicates that presence of both malignant and benign mammographic MCs is associated with poor prognostic factors of breast cancer, and could serve as indicators of aggressive tumor growth.

This study found a positive relationship between the presence of MCs and over-expression of HER-2. HER-2 is a valuable therapeutic and prognostic marker in primary breast carcinomas [10]. It plays a significant role in the HER family of receptors, normally involved in regulating breast growth and development. Over-expression of HER-2 proto-oncogene, called c-erbB-2, is associated with breast cancer [11]. This gene is amplified in approximately 20 to 30% of breast cancers and is associated with aggressive tumor behaviour [12]. Wang et al. [10] conducted a retrospective study of 152 patients, and found MCs were more common in carcinomas with HER-2 over-expression at a prevalence of 61.6% compared to those without HER-2 at 35.4%. Similarly, Seo et al. [13] also found an association between mammographic MCs and HER-2 over-expression in 498 patients. Our study further confirms a greater prevalence of MCs (52.9%) among tumors over-expressing HER-2, compared to tumors without HER-2 amplification (33.8%). This is further reinforced by the figures, showing presence of MCs for a HER-2 positive patient (Figure 1) and a complete absence of MCs for a HER-2 negative patient (Figure 2). However, unlike Seo et al. [13] who found more MCs in patients with HER-2 over-expression under the age of 50, our results showed that the presence of MCs was independent of patient age (p = 0.100). Our study cohort was also twice (n = 937) as large as the population assessed by Seo et al. (n = 498). Given the strong association, MCs could serve as early indicators of HER-2 over-expression, warranting further molecular investigation into their relationship.

Hormone receptor status is useful for its prognostic significance and treatment planning in patients with advanced breast cancer. Previous studies have investigated the association between MCs and hormone receptor status with variable results. Griniatsos et al. [14] found an increased number of patients with both estrogen and progesterone receptor positive tumors presented with mammographic MCs. Similarly, Karamouzis et al. [15] also found MCs in over 65% of ER positive, and over 46% of PR positive tumors. On the contrary, Ferranti et al. [16] found an inverse relationship between mammographic MCs and hormone receptor positive lesions. The prognostic significance of ER and PR expression has been a matter of debate for many years. However, current available evidence suggests that ER/PR negative tumors have a worse prognosis altogether [17]. Similar to Gajdos et al. [18], our study showed that the presentation of MCs is independent of hormone receptor status.

Furthermore, strong correlations between MCs and tumor grade, tumor histology, and breast density highlight the prognostic significance of these calcium deposits. Our results showed that a higher prevalence of MCs was found among patients with high grade tumors than those with low grade tumors. This result reinforces previous studies, such as that by Palka et al. [9], which showed a strong relationship between MCs and high grade lesions. Conversely, Dinkel et al. [19] found this correlation to be poor and inconclusive.

To better understand these associations, the relationship between mammographic breast densities was compared to the presence of MCs. The physical composition of the breast varies, with different proportions of fat, connective tissue, ductal and lobular elements contributing to differences in mammographic breast density. The greater the number of fibroglandular tissue, the higher the category of breast density. Our results confirmed those of Skandalis et al. [20], who found elevated levels of MCs in patients with heterogeneously dense breasts and high tumor grades. We found MCs to be significantly associated with heterogeneous breast densities, with a high prevalence of fibroglandular tissue.

The link between tumor grade and breast density highlights some molecular factors giving rise to MCs and contributing to tumorigenesis. Tabar and Dean [21] propose that high-grade ductal carcinomas undergo a process called neoductogenesis, promoting vascular invasion, with excessive lymphatic and hematogenous spread, leading to a worse tumor prognosis. A high prevalence of fibroglandular breast tissue can lead to increased accumulation of versican, a proteoglycan associated with high tumor grade and invasive disease in patients with high breast densities and mammographic MCs [20].

Approximately 90% of ductal carcinoma in situ (DCIS) appear as MCs, 40% of which progress to an invasive breast cancer. Among invasive carcinomas, our study further discovered a greater prevalence of MCs in multifocal invasive disease (47.9%) than unifocal invasive disease (36.1%). Tot et al. [22] studied the influence of tumor focality on breast cancer survival, and found the highest ten year survival rate to be amongst patients with unifocal tumors. Multifocal tumors serve as poor prognostic parameters in breast cancer, and their strong association with MCs further reinforces the role of MCs as poor prognostic indicators of breast cancer.

There was no significant outcome difference between patients with MCs and those without MCs. Recurrence was measured using a 5 year recurrence end-point, and there was no statistical association between having MCs on mammography and recurrence pattern (p = 0.258).

These results could also be affected by our study limitations. Our study population was limited to St. Michael’s hospital, whereas a multi-site analysis would have allowed for a more rigorous analysis. We also conducted a retrospective chart review, where missing data on electronic charts could not be included in the study. Hence, as seen in Table 1, numbers for all variables vary due to incomplete information recorded on patient charts. Inclusion of additional biomarkers, such as p53 and further genetic analysis would have further improved our understanding of the relationship between MCs and breast cancer.

Furthermore, MCs smaller than 130 μm were not visible on our digital mammogram [23]. In these circumstances, histological examination of breast tissue can reveal smaller MCs that were missed in mammography. To test for MCs that were missed in mammograms, we compared the presence of MCs among pathology and mammography reports for each patient. Table 3 further depicts these discrepancies. Our study showed that 52.2% of MCs that were absent on mammograms were visible under a histological examination. Hence, our results were influenced by the size limitations of mammograms. Also, we did not examine the chemical composition of MCs, which would have been useful for an accurate understanding of their role in breast tumorigenesis.

## Conclusions

In summary, this study is the largest correlation analysis performed to date, investigating the association of any mammographic MCs in breast cancer patients with variables of breast cancer. Based on the strong associations between MCs and poor prognostic indicators of breast cancer, such as HER-2 over-expression, high tumor grade, prevalence of fibroglandular tissue, and multifocal disease, it can be suggested that MCs are strongly associated with breast cancer variables that lead to a poor prognosis. Based on these results, MCs warrant closer attention and follow-up. There is also a need for developing improved screening methods to detect smaller MCs that might otherwise be missed on screening mammograms. Since biological distinctions between subtypes of breast cancers likely reflect differences in the pathways of tumor development and disease prognosis, future studies should investigate the molecular pathways interconnecting MC genesis with breast tumorigenesis.

## Abbreviations

MC: Microcalcifications
ER: Estrogen receptor
PR: Progesterone receptor
HER-2: Human epidermal growth factors receptor 2
SMH: St. Michael’s Hospital
IHC: Immunohistochemistry
FISH: Fluorescent in sity hybridization
DISH: Dual in situ hybridization

## References

[1] Breast cancer statistics. [http://www.cancer.ca].

[2] Tabar L, Duffy SW, Vitak B, Chen HH, Prevost TC (1999). The natural history of breast carcinoma: what have we learned from screening?. Cancer.

[3] Bellahcene A, Castronovo V (1995). Increased expression of osteonectin and osteopontin, two bone matrix proteins, in human breast cancer. Am J Pathol.

[4] Nalawade YV (2009). Evaluation of breast calcifications. Indian J Radiol Imaging.

[5] Bansal GJ, Thomas KG (2011). Screen-detected breast cancer: does presence of minimal signs on prior mammograms predict staging or grading of cancer?. Clin Radiol.

[6] Castronovo V, Bellahcene A (1998). Evidence that breast cancer associated microcalcifications are mineralized malignant cells. Int J Oncol.

[7] Morgan MP, Cooke MM, McCarthy GM (2005). Microcalcifications associated with breast cancer: an epiphenomenon or biologically significant feature of selected tumors?. J Mammary Gland Biol Neoplasia.

[8] Zunzunegui RG, Chung MA, Oruwari J, Golding D, Marchant DJ, Cady B (2003). Casting-type calcifications with invasion and high-grade ductal carcinoma in situ: a more aggressive disease?. Arch Surg.

[9] Palka I, Ormandi K, Gaal S, Boda K, Kahan Z (2007). Casting-type calcifications on the mammogram suggest a higher probability of early relapse and death among high-risk breast cancer patients. Acta Oncol.

[10] Wang Y, Ikeda DM, Narasimhan B, Longacre TA, Bleicher RJ, Pal S, Jackman RJ, Jeffrey SS (2008). Estrogen receptor-negative invasive breast cancer: imaging features of tumors with and without human epidermal growth factor receptor type 2 overexpression. Radiology.

[11] Yarden Y (2001). Biology of HER2 and its importance in breast cancer. Oncology.

[12] Hynes NE, Stern DF (1994). The biology of erbB-2/neu/HER-2 and its role in cancer. Biochim Biophys Acta.

[13] Seo BK, Pisano ED, Kuzimak CM, Koomen M, Pavic D, Lee Y, Cole EB, Lee J (2006). Correlation of HER-2/neu overexpression with mammography and age distribution in primary breast carcinomas. Acad Radiol.

[14] Griniatsos J, Vassilopoulos PP, Kelessis N, Agelatou R, Apostolikas N (1995). The prognostic significance of breast tumour microcalcifications. Eur J Surg Oncol.

[15] Karamouzis MV, Likaki-Karatza E, Ravazoula P, Badra FA, Koukouras D, Tzorakoleftherakis E, Papavassiliou AG, Kalofonos HP (2002). Non-palpable breast carcinomas: correlation of mammographically detected malignant-appearing microcalcifications and molecular prognostic factors. Int J Cancer.

[16] Ferranti C, Coopmans DYG, Biganzoli E, Bergonzi S, Mariani L, Scaperrotta G, Marchesini M (2000). Relationships between age, mammographic features and pathological tumour characteristics in non-palpable breast cancer. Br J Radiol.

[17] Hayes DF (2011). Disease related indicators for a proper choice of adjuvant treatments. Breast.

[18] Gajdos C, Tartter PI, Bleiweiss IJ, Hermann G, Csepel J, Estabrook A, Rademaker AW (2002). Mammographic appearance of nonpalpable breast cancer reflects pathologic characteristics. Ann Surg.

[19] Dinkel HP, Gassel AM, Tschammler A (2000). Is the appearance of microcalcifications on mammography useful in predicting histological grade of malignancy in ductal cancer in situ?. Br J Radiol.

[20] Skandalis SS, Labropoulou VT, Ravazoula P, Likaki-Karatza E, Dobra K, Kalofonos HP, Karamanos NK, Theocharis AD (2011). Versican but not decorin accumulation is related to malignancy in mammographically detected high density and malignant-appearing microcalcifications in non-palpable breast carcinomas. BMC Cancer.

[21] Tabar L, Dean PB (2008). Thirty years of experience with mammography screening: a new approach to the diagnosis and treatment of breast cancer. Breast Cancer Res.

[22] Tot T, Gere M, Pekar G, Tarian M, Hofmever S, Hellberg D, Lindguist D, Chen TH, Yen AM, Chiu SY, Tabar L (2011). Breast cancer multifocality, disease extent, and survival. Hum Pathol.

[23] Bick U, Diekmann F (2007). Digital mammography: what do we and what don’t we know?. Eur Radiol.

